# Drought Response in the Transcriptome and Ionome of Wild and Domesticated *Lablab purpureus* L. Sweet, an Underutilized Legume

**DOI:** 10.1002/pei3.70027

**Published:** 2025-01-19

**Authors:** Anastasia Kolesnikova, John Hammond, Mark A. Chapman

**Affiliations:** ^1^ School of Biological Sciences University of Southampton Southampton UK; ^2^ School of Agriculture, Policy and Development University of Reading Reading UK

**Keywords:** drought, dual purpose, elemental, food/forage species, ionome, lablab, underutilized crop

## Abstract

Hunger remains a prevalent issue worldwide, and with a changing climate, it is expected to become an even greater problem that our food systems are not adapted to. There is therefore a need to investigate strategies to fortify our foods and food systems. Underutilized crops are farmed regionally, are often adapted to stresses, including droughts, and have great nutritional profiles, potentially being key for food security. One of these crops, *Lablab purpureus* L Sweet, or lablab, is a legume grown for humans or as fodder and shows remarkable drought tolerance. Understanding of lablab's molecular responses to drought and drought's effects on its nutritional qualities is limited and affects breeding potential. Using transcriptomics at three time points, changes in gene expression in response to drought were investigated in wild and domesticated lablab. The effect of drought on the elemental profile of lablab leaves was investigated using ionomics to assess drought's impact on nutritional quality. Differences in drought response between wild and domesticated lablab accessions were revealed, which were mainly due to differences in the expression of genes related to phosphorus metabolic response, cell wall organization, and cellular signaling. The leaves of wild and domesticated lablab accessions differed significantly in their elemental concentrations, with wild accessions having higher protein, zinc, and iron concentrations. Drought affected the concentration of some elements, with potential implications for the use of lablab under different environments. Overall, this study is an important first step in understanding drought response in lablab with implications for breeding and improvement of drought‐tolerant lablab.

## 1 Introduction

The UN Sustainable Development Goals (SDGs) 2 and 3 focus on eradicating hunger and ensuring good health and well‐being for the world (Naeem et al. 2020). This requires a concentrated effort in combatting undernutrition. Undernutrition arises when people cannot meet their macronutrient and micronutrient requirements and occurs in both developed and developing countries (Li, Yadav, and Siddique 2020). In 2017, about 800 million people were chronically hungry, without getting enough calories, and approximately 2 billion people experience hidden hunger—an inadequate intake of key micronutrients (Gödecke, Stein, and Qaim 2018; Weffort and Lamounier 2023). This is an extremely pressing issue for countries in Sub‐Saharan Africa, as it has been one of the few regions of the world that has been unable to overcome chronic food deficits (Pingali 2012).

With millions of people already food insecure, climate change adds an additional challenge to achieving food security, with the projected increase of the number of additional people at risk of hunger being 5 to 170 million (Schmidhuber and Tubiello 2007). An increase in global temperatures will also directly affect agricultural production; increases in total annual precipitation and extreme weather events may severely reduce yields for crops like maize in temperate regions (Rosenzweig et al. 2002). On the other hand, droughts may increase in frequency in other parts of the world, leading to a decrease in crop yield, even in developed countries like the USA (Leng and Hall 2020). Drought can also negatively affect the nutritional quality of plant foods.

It is therefore important for any intervention to alleviate global hunger to ensure that it can withstand an unpredictable, changing climate, and provide adequate macro and micronutrients for the growing population. Our current food system is not equipped to deal with these challenges. Out of the approximately 30,000 edible species of plants, we cultivate only 7000 for consumption, with three (rice, wheat and maize) providing an estimated 42% of our calories (Elert 2014; Muthamilarasan, Singh, and Prasad 2019). The productivity of these crops is predicted to significantly decline with each degree rise in global temperature; additional pressures like erratic rainfall may lead to an increase in irrigation demand which is challenging to meet in some parts of the world (Muthamilarasan, Singh, and Prasad 2019; Zhao et al. 2017). Therefore, urgent action is needed to ensure that food security can be achieved in a changing climate and that the SDGs can be met.

There are various methods that can help to improve food security, food system diversification being one of them. Diversification involves exploring growing new crops, intercropping or generating new end products (Kremen, Iles, and Bacon 2012). This helps to boost resilience in agriculture, as various species perform different functions in different environments, which is key for agriculture in unstable environments due to climate change (Mustafa, Mayes, and Massawe 2019).

There has been a push for research on using underutilized crops for diversification. Underutilized crops are wild and domesticated plant species which are not grown or consumed globally and have received limited research attention (Minde, Matemu, and Venkataramana 2022; Prasad 2020). These crops can have certain advantages over more domesticated crops, which include better ability to withstand stresses, higher micronutrient concentrations, and in the case of legumes, the ability to fix nitrogen, making them useful for intercropping and low‐input production systems (Alhassan and Egbe 2014; McMullin et al. 2021; Prasad 2020). Additionally, these crops can strengthen local food systems and identities, helping to empower marginalized communities and preserve indigenous knowledge (Chivenge et al. 2015; Prasad 2020). With the spread of staple crops during the 20th century, many underutilized crops have been abandoned, and so further measures may be needed to help these crops achieve their full potential (Mustafa, Mayes, and Massawe 2019; Shorinola et al. 2024).

*Lablab purpureus* L. Sweet (Fabaceae, 2n = 22), commonly known as lablab or hyacinth bean, is a multifunctional legume which can be used as a pulse, vegetable, livestock feed, green manure, and for decorative and medicinal purposes (Kongjaimun et al. 2023; Maass 2016; Naeem et al. 2020). Different varieties can be grown as either an annual or a perennial crop (Sherasia, Garg, and Bhanderi 2018). The genus *Lablab* is monotypic and *L. purpureus* L. Sweet contains three subspecies: ssp. *purpureus*, which is primarily cultivated in Africa and India, ssp. *bengalensis*, which is primarily cultivated in Asia, and ssp. *uncinatus*, which is the wild form spread across East Africa (Minde, Matemu, and Venkataramana 2022). There are two gene pools in lablab, differing in the number of seeds per pod, with wild and domesticated types in both, indicating two domestications (Njaci et al. 2023).

Lablab is a genetically diverse and versatile crop, as it can be grown in a range of climates and soil types, tolerating a wide range of soil pH, rainfall, and temperatures, and is well‐adapted to drought (Sherasia, Garg, and Bhanderi 2018). It possesses a great nutritional profile, being high in protein, iron, phosphorus and zinc, which is useful in combatting nutrient insufficiency (Letting, Venkataramana, and Ndakidemi 2021). However, it also contains anti‐nutritional compounds, such as tannins and trypsin inhibitors (Maass 2016). Other challenges with the widespread growth of lablab include lack of disease resistance, a need for cultivars with better salinity and drought tolerance and improved marketability (Letting, Venkataramana, and Ndakidemi 2022). Despite its ability to tolerate stresses like drought, cultivars with higher tolerance could still be developed (Missanga, Venkataramana, and Ndakidemi 2021). There has been an interest in developing breeding tools for lablab, resulting in the publication of a chromosome‐level assembly of its genome and resequencing data from a range of wild and domesticated accessions (Njaci et al. 2023).

Plants may use multiple strategies to respond drought stress, including drought escape, drought avoidance, drought tolerance, and drought recovery (Fang and Xiong 2015). Drought tolerance is defined as the ability to change physiological activity to prevent plant dehydration and continue metabolism at low water levels (Manavalan et al. 2009). Response to drought is a complex network of biochemical and physiological processes. Legumes' ability to tolerate drought is heavily species specific (Daryanto, Wang, and Jacinthe 2015) and lablab's response to water stress has been investigated through metabolic and genetic approaches (see Data S1 and summarized below).

Upon sensing drought, legumes may respond by controlling water loss through either regulating stomata or through osmotic adjustment (Daryanto, Wang, and Jacinthe 2015). In lablab, a reduction in water content in stressed plants, a decrease in plant height and decreased stomatal conductance have been observed; drought tolerant genotypes have lower transpiration rate, higher chlorophyll content/stability and a lower proline content (Guretzki and Papenbrock 2014; Sawardekar et al. 2020). Another aspect of drought response in legumes are redox processes, as stress increases the production of ROS (Cruz de Carvalho 2008). Metabolic studies in lablab have shown an increase in antioxidants, antioxidant enzymes, and osmolytes like proline and total soluble sugars, with varying proportions in the roots and leaves (D'souza and Devaraj 2011; Kokila, D'souza, and Devaraj 2014).

Gene expression changes under water stress have been investigated in two Chinese lablab varieties, MEIDOU 2012 and NANHUI 23 (Wang et al. 2018; Yao et al. 2016, 2013). Comparing stressed to non‐stressed plants, 27 transcription factors were differentially expressed, including a R2R3‐MYB factor, *LpMYB1* (Wang et al. 2018; Yao et al. 2016). The most recent genetic study identified 43 lablab transcripts differentially expressed under drought and salinity stress (Srinivasa and Devaraj 2021). Next‐generation sequencing approaches, such as transcriptomics, may be able to provide wider understanding of genes involved in drought stress than techniques that have been previously used to investigate drought tolerance in lablab. Transcriptomics has been used to investigate drought‐related gene expression changes in soybean, lentil, chickpea, and common bean, however, transcriptomic studies in underutilized crops like lablab are lacking (Jha, Bohra, and Nayyar 2020). Transcriptomics resources in underutilized crops have helped identify breeding loci and identified genes involved in developmental responses, but work on understanding of stress responses and identifying candidate genes for improvement is lacking (Chapman 2015; Suranjika et al. 2022). There is a need for transcriptomic investigations for drought response in lablab to identify potential genes of interest for further improvement of the crop. Additionally, it is known that wild relatives may harbor adaptive genetic diversity which can be used for breeding purposes (von Wettberg, Davis, and Smýkal 2020). Several crop wild relatives are known to be tolerant of stress conditions such as salinity (Abdul Aziz and Masmoudi 2023); *Strophostyles helvola* (L.) Elliott, a wild relative of the common bean, is one of the few cases where a transcriptomics analysis has been used to understand salinity tolerance (Villanueva et al. 2023). However, drought tolerance of crop wild relatives, especially underutilized crops, has not yet been investigated through transcriptomics.

Environmental stresses like drought can also impact on the nutritional quality of the crop, which is another key challenge for food security. Previous studies have shown that key legumes, such as chickpeas, cowpea, lentil, soybean and mung bean, have lower protein concentrations under drought and heat stress (Jha et al. 2024). In addition, under drought, lentil micronutrient concentrations decreased, with lower amounts of key minerals like calcium, iron, and zinc (Sehgal et al. 2018). Whether analogous changes in nutrition happen in more drought‐tolerant crops like lablab is an unanswered question that can be addressed by techniques such as ioniomics. Ionomics is a technique that quantifies the mineral nutrient and trace element composition of tissue, allowing to quantify key nutrients within a sample (Salt, Baxter, and Lahner 2008).

This study aims to increase the understanding of lablab drought response by using transcriptomics at three time points to uncover the molecular mechanisms of drought response in lablab including comparing wild and domesticated lablab. We applied a low water stress that was insufficient to meet plant needs (Redmond 2002) which we determine to be a physiological drought because we observed slower growth and additional phenotypic responses. Additionally, to investigate the effect of drought on lablab nutrition, we carried out a nutritional analysis of wild and domesticated lablab leaves which have or have not been subjected to drought.

## 2 Materials and Methods

### 2.1 Plant Growth

Ten accessions (five wild, five domesticated landraces; Table 1) were selected for the experiment and supplied by the International Livestock Research Institute (Nairobi, Kenya). These accessions come from East Africa, where droughts have been increasing in frequency and below‐average rainfall has occurred almost yearly since 2008 (Gebremeskel Haile et al. 2019). Seeds from each accession were planted into five pots per accession (diameter = 19 cm, height = 16 cm) containing a 50:50 ratio of medium (2.0–5.0 mm) Sinclair vermiculite and Seed and Modular + Sand Medium Nutrient ICL Levington Advance growing media. Up to three seeds were planted per pot and if more than one germinated, the plants were separated into different pots. One domesticated accession did not germinate and so is not considered further. In total 56 plants germinated and were used for the experiment. These were grown on a bench in the University of Southampton Glasshouse (20°C day, 18°C night, although max temperature sometimes reached 23°C; 12 h daylength; watered once a day for 5 min by bench flooding). All seedlings were grown for 24 days under these conditions before the drought treatment started.

**TABLE 1:** The wild (accessions starting with W) and domesticated (accessions starting with D) lablab accessions used in the experiment, with the accessions used in RNAseq^a^, and the total number of plants that germinated in the experiment for each accession. Seeds were obtained from the International Livestock Research Institute. Respective ILRI Genebank links are provided in Data S2.

Accession	Population	Origin	Total no. of plants
D14411^a^	Domesticated	Kenya	8
D14419	Domesticated	Zimbabwe	2
D21049^a^	Domesticated	Mozambique	5
D21085^a^	Domesticated	Zambia	7
W21048	Wild	South Africa	6
W21081^a^	Wild	Uganda	7
W24749	Wild	Zimbabwe	5
W24750^a^	Wild	Kenya	9
W24778^a^	Wild	Zimbabwe	7

^a^ Denotes that this accession was used for RNA‐sequencing.

Plants from each accession were divided into two groups and assigned a treatment by using a random number generator (https://www.random.org/). Those selected for the control treatment were simply maintained under the same conditions, whereas those selected to receive a drought treatment were placed on a dish that prevented water from the bench entering during watering. Because the plants from different accessions were of different height, leaf size and biomass, we did not set a specified length of time to perform the stress, as this could result in the sampling of plants that differ in physiology and maturity and therefore may lead to a “pot effect” rather than obtaining genuine changes with stress (Moshelion et al. 2024). Instead, each plant was monitored daily until multiple symptoms of stress (drying or wilting of lower leaves, curling or yellowing of newer leaves, leaf drooping) were observed (Figure S1), as wilting and dropping of leaves are known to be symptoms of drought stress, and until soil water moisture dropped under 15%, as measured by SM150T Soil Moisture Sensor with the HH2 Moisture Meter (Delta‐T Devices, Cambridge, UK) (Akello et al. 2023; Moshelion et al. 2024; Missanga, Ndakidemi, and Venkataramana 2023). At this point the dish was removed, and the plant was rewatered from the next day onwards by bench flooding. Plants continued to be watered for around 4 weeks post‐stress, until they had made a full recovery, which was quantified by the appearance of new leaves.

### 2.2 RNAseq Sampling and Analysis

Samples for RNAseq were taken at three time points—the day before the water was withheld (“pre‐drought”), when plants were proclaimed stressed (“drought”) and around 4 weeks after stress (“recovery”), when they had recovered, with the pre‐drought samples being a time course control (Spies and Ciaudo 2015). Sampling was done at the same time (11:30 am) to reduce differences in expression due to circadian rhythm. One larger or two smaller leaves was sampled from each plant, placed in a 1.5 mL Eppendorf tube and immediately placed in dry ice. Leaf samples were stored at −80°C until RNA extraction. Plants chosen for RNAseq were selected to be of similar developmental stage (within accession) before the treatments began.

RNA extraction was carried out using a QIAGEN RNeasy Plant Mini Kit, according to the “Plant and Fungi” protocol detailed in the RNeasy Mini Handbook, with the Optional On‐Column DNase Digestion. Three wild and three domesticated accessions were selected (Table 1) and one plant from each time point was used for extraction (total 18 samples; our focus was how the subspecies responded (not each accession) therefore accessions are treated as replicates within each treatment/timepoint). RNA quality was checked using a Nanodrop Spectrophotometer to ensure sample concentration was > 50 ng/μl, and OD ratios 260/280 and 260/230 were around 2.0. RNA was shipped on dry ice to Novogene (Cambridge, UK) and at least 6GB of paired end data per sample, 2 × 150 bp, was generated on an Illumina Novaseq.

Sequencing reads were quality checked using FASTQC v9.0.4 (Andrews 2010). They were then trimmed using Trimmomatic v0.36 (Bolger, Lohse, and Usadel 2014), removing TruSeq3 adapters, with a sliding window of 4:15, and minimum leading and trailing base qualities set at 5. The minimum read length was set at 72. A FASTA file containing the lablab genome and a GTF file containing corresponding annotations including transposable elements were obtained from https://hpc.ilri.cgiar.org/~bngina/lablab_longread_sequencing_March_2022/ (Njaci et al. 2023). These were used to construct a genome index in STAR v2.7.10a (Dobin et al. 2013) with the default settings. Paired‐end reads were aligned to the genome using STAR with default settings, and the output sorted by coordinate. RSEM v1.3.3 (Li and Dewey 2011) was then used to calculate expression of reads, with the aligner set to STAR. Isoform expected counts were obtained and rounded in R, as suggested by Li and Dewey (2011) and only genes which had more than one read mapped were included. For differential expression (DE) analysis, three datasets were created:

- Expected counts for samples before and during drought (i.e., pre‐drought vs. drought).
- Expected counts for samples before drought and after recovery (i.e., pre‐drought vs. recovery).
- Expected counts for samples during drought and after recovery (i.e., drought vs. recovery).

DE analysis was carried out using DESeq2 v1.37.6 (Love, Huber, and Anders 2014) in R v4.2.1 (R Core Team, 2022), as done in similar studies (Li et al. 2022; López, Pineda, and Alamillo 2020). The data was checked for outliers using a PCA (Figure 3) using the stats and ggplot2 (Wickham 2016) packages.

DE analysis compared each pair of timepoints to find all genes which are DE between time points and compared wild and domesticated samples at each pair of timepoints. We also identified genes with a significant interaction (i.e., those with a different response to drought between the wild and domesticated populations). A volcano plot was created for each result using the EnhancedVolcano package v3.15 (Blighe, Rana, and Lewis 2023). Results were further annotated with known orthologues from *Arabidopsis thaliana* , which were obtained by a BLAST (v.2.14.1) search of lablab gene sequences against cds available from TAIR10 genome release (https://www.arabidopsis.org/) (Berardini et al. 2015). Genes with an adjusted *p* < 0.05 were deemed DE and used in Gene Ontology analyses using agriGO v2.0 (http://systemsbiology.cau.edu.cn/agriGOv2/classification_analysis.php?category=Plant&&family=Fabaceae) (Tian et al. 2017). A Singular Enrichment Analysis was carried out for the Fabaceae group using *Arabidopsis* annotations against the suggested background. GO terms and their FDR‐corrected P values were then summarized and visualized using REVIGO (http://revigo.irb.hr/), with the resulting list size set as small (0.5), obsolete terms removed, and the species set to *Cicer arietinum* , the representative legume available. Semantic similarity was measured using SimRel, with the default method (Supek et al. 2011).

Differentially expressed genes which overlapped between the three comparisons above were obtained using Venny (Oliveros 2007), and their known orthologues were submitted for another agriGO and REVIGO analysis. Overlapping GO terms were also obtained and submitted for a REVIGO analysis. For one GO term, response to osmotic stress (GO:0006970), gene expression dynamics were investigated in more detail. The original RSEM counts for the genes corresponding to this term were obtained and the average expression values at each stage for wild and domesticated accessions were plotted in R using ggplot2.

### 2.3 Nutrient Analysis Sample Preparation and Analysis

For nutrient analysis, young leaves from the top of the plant were collected from control and recovered plants that had been growing for 80 days. In a few cases limited fresh leaves were available (see Results) and so some older leaves were included. The leaves were weighed, dried in an oven at 60°C for 48 h and re‐weighed to calculate water content and then ground to a fine powder.

Total nitrogen was determined using a LECO CHN628 series Elemental Analyzer. Dried lablab leaf samples weighing approximately 0.1 g were encapsulated as small balls with tin foil cups and then loaded into the LECO autoloader. Resulting nitrogen % content was converted to protein by multiplying nitrogen content by factor 5.7 and results then reported as % protein concentration (Yeoh and Wee 1994).

Subsamples (~0.50 g DW) of leaf were digested using a microwave system Ethos Easy 44‐ max microwave digestor platform with a 44‐vessel rotor (Analytix UK, Tyne & Wear, UK) to determine leaf nutrient concentrations based on Thomas et al. (2016). Leaf material was digested in 8 mL 70% Trace Analysis Grade HNO_3_ and 2 mL Milli‐Q water (18.2 MΩ cm; Fisher Scientific UK Ltd., Loughborough, UK), with microwave settings as follows: power = 1400 W, temp = 140°C, pressure = 2 MPa, time = 45 min. Two operational blanks were included in each digestion run. Duplicate samples of certified reference material (CRM) of leaf (Cabbage IPE 898, IPAE Wageningen, NL) were included in every digestion run. Following digestion, each tube was made up to a final volume of 50 mL by adding Milli‐Q water and transferred to a 50 mL universal tube (Sarstedt Ltd., Nümbrecht, Germany) and stored at room temperature.

Leaf digestates were diluted 1‐in‐5 using Milli‐Q water prior to elemental analysis. The concentrations of elements were obtained using inductively coupled plasma‐optical emission spectrometry (ICP‐OES; Avio 550 Max ICP Optical Emission Spectrometer, Perkin Elmer, UK).

Phenotypic data (number of days to show stress and leaf water content) were analyzed in R. Number of days to show stress was checked for normality using a Wilks–Shapiro test (Shapiro and Wilk 1965) and a Q‐Q plot and a one‐way ANOVA was used to compare the wild and domesticated groups. The dry weight and water content was also subjected to the same normality testing and then a two‐way ANOVA was run to compare wild vs. domesticated and control vs. stress treatment and their interaction.

Nutrient data was tested for normality as above and transformed where necessary based on skewness using either the Log_10_ or the square root transform were applied (Data S7). A two‐way ANOVA was run on each element to compare wild versus domesticated and control versus stress treatment and their interaction.

## 3 Results

### 3.1 Wild Accessions Had Lower Biomass, But Higher Fe, Zn and Cu Concentrations Compared to Domesticated Accessions

**FIGURE 1:**
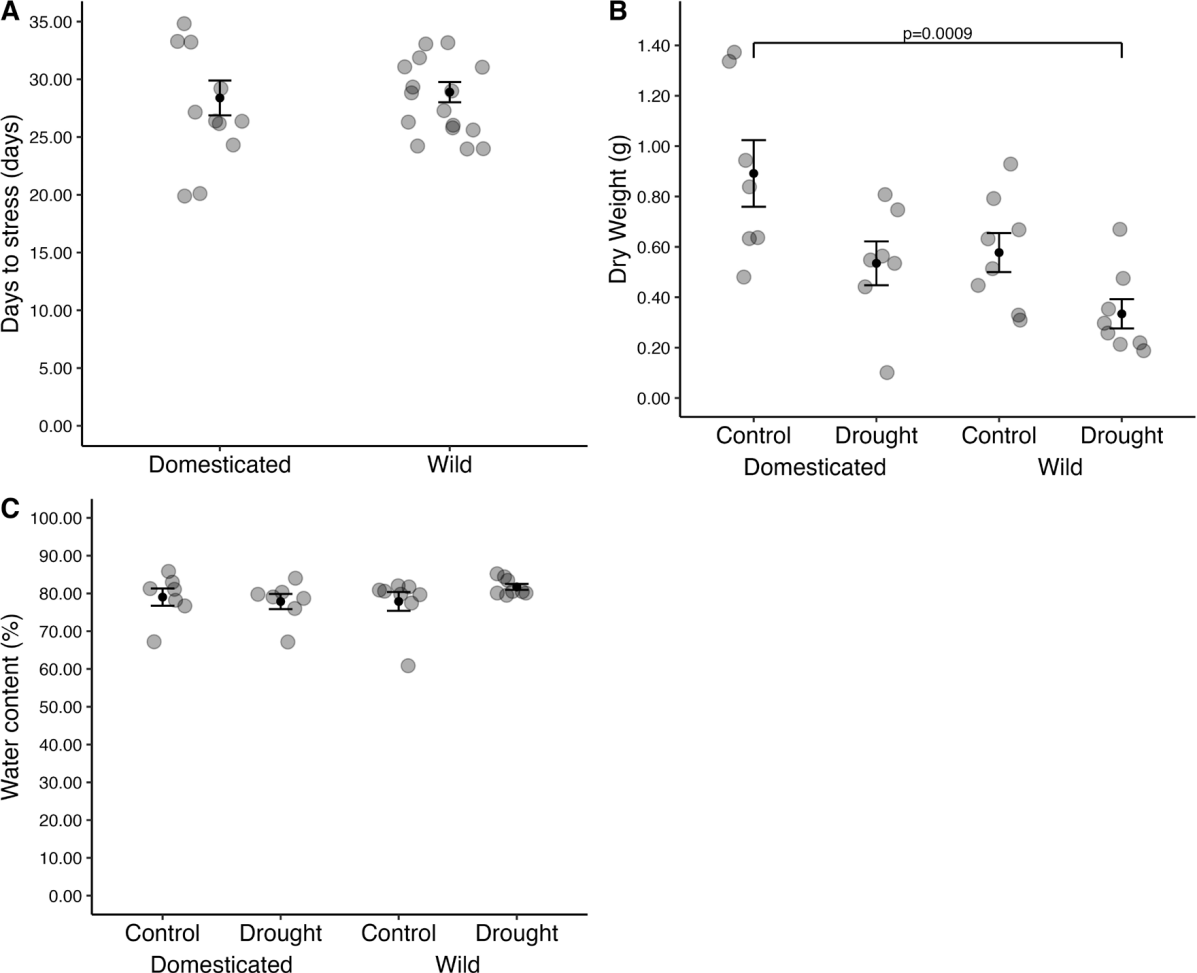
Phenotypic parameters including mean days to visual signs of stress (*n* wild = 18; *n* domesticated = 13) (A), mean leaf dry mass (*n* wild = 16, *n* domesticated = 14), (B) mean leaf water content, (C) were measured in wild and domesticated lablab accessions, under drought or control (well‐watered) conditions. Plants were grown in pots for 24 days in a glasshouse before water was withdrawn to initiate drought stress. Error bars represent ± standard error of the mean. Significant comparisons found by a TukeyHSD test and their p‐values are represented by a bracket.

Overall, the average number of days it took for both wild and domesticated accessions to show symptoms of drought stress were not significantly different with wild accessions taking 28.8 days on average to become visibly stressed and domesticated accession taking 28.5 days (one‐way ANOVA F = 0.095, *p* = 0.761; Figure 1a). One accession of each (W21081 and D21085) took over 4 weeks to become visibly stressed (Data S3).

The leaf biomass was significantly different depending on both the treatment (*F* = 10.841, *p* = 0.002; Figure 1b) and the domestication status (*F* = 8.147, *p* = 0.008) without a significant interaction (*F* = 0.395, *p* = 0.535).

The mean water content of lablab leaves was 77.9% and 81.7% for domesticated and wild lablabs that underwent drought respectively (Figure 1c). The mean water content for domesticated and wild accessions under control conditions was 79.0% and 77.9% respectively (Figure 1c; Data S4). There was no significant effect of domestication status (*F* = 0.408, *p* = 0.529) or treatment (*F* = 0.598, *p* = 0.446) and no interaction (*F* = 1.518, *p* = 0.229) on the water content of lablab leaves.

**FIGURE 2:**
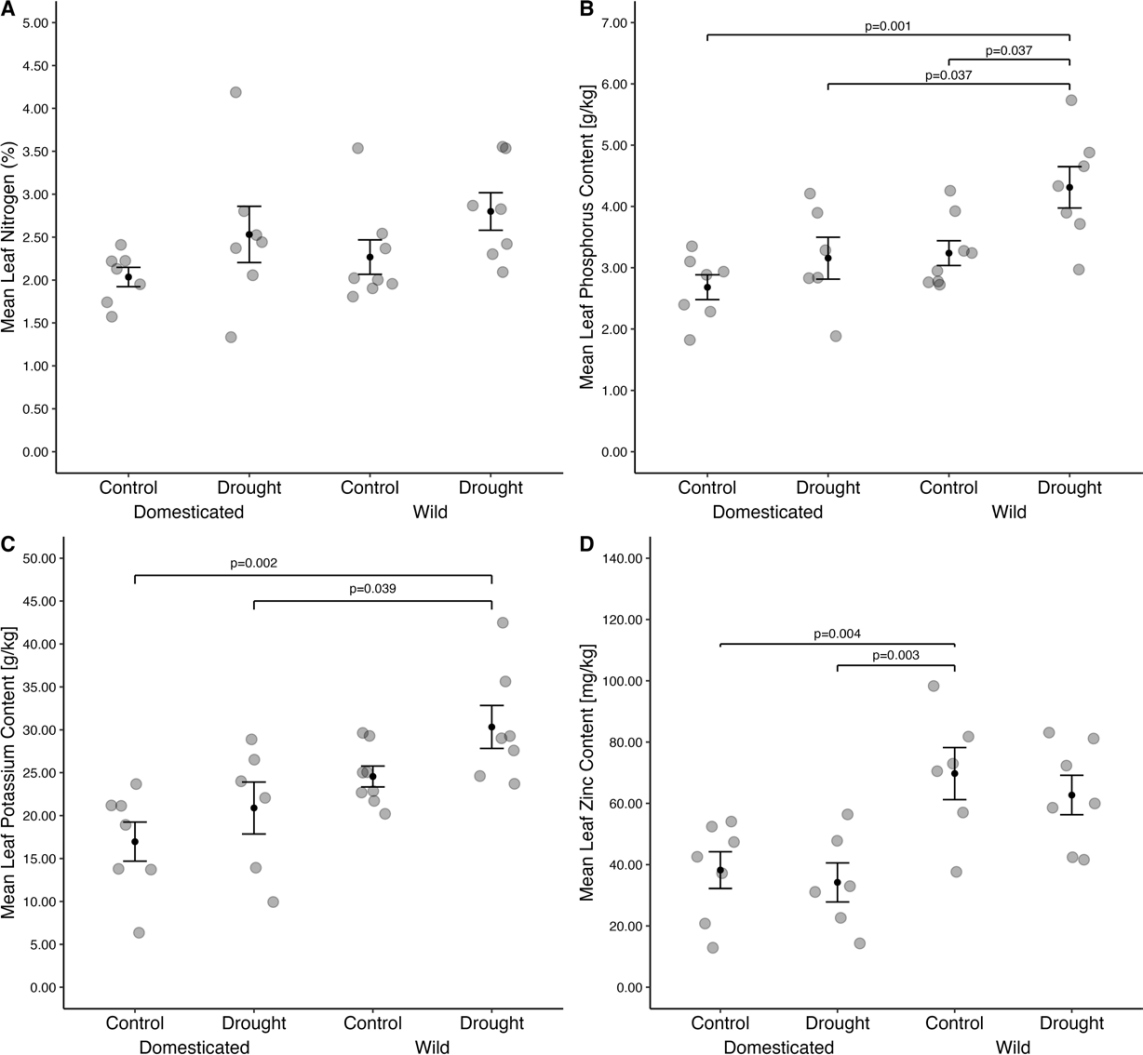
Leaf nitrogen (A) phosphorus (B) potassium (C) and zinc (D) concentrations in wild and domesticated lablab accessions grown under drought (dark gray bars) or control (well‐watered; light gray) conditions. Plants were grown in pots for 24 days in a glasshouse before water was withdrawn to initiate drought stress. Bars represent means (*n* wild = 16, *n* domesticated = 14) ± standard error of the mean. Significant comparisons found by a TukeyHSD test and their p‐values are represented by a bracket.

The concentration of 16 elements was measured in the leaves of wild and domesticated accessions at the same timepoint, from both well‐watered and droughted plants (Figure 2; Data S5–S6; Figures S2–S5).

The concentration of potassium (*F* = 14.330, *p* = 0.001), phosphorus (*F* = 9.550, *p* = 0.005), copper (*F* = 7.217, *p* = 0.013), iron (*F* = 12.267, *p* = 0.002), and zinc (*F* = 17.173, *p* = 0.000) were significantly different between wild and domesticated accessions. Wild accessions had higher concentrations of iron, copper, and zinc compared to domesticated accessions. Potassium (*F* = 4.820, *p* = 0.038), phosphorus (*F* = 8.607, *p* = 0.007) and nitrogen (*F* = 4.909, *p* = 0.036) concentrations were significantly affected by drought treatment. The plants that received drought treatment had higher concentrations of these nutrients than the control plants. Sulfur concentration had a significant interaction (*F* = 14.005, *p* = 0.001).

### 3.2 RNAseq Demonstrates Different Transcriptional Responses Between Domesticated and Wild Accessions

An average of 22.1 M reads (± 2.2 M SD) were obtained from each sample, of which 3.3% (± 0.3% SD) on average was removed during quality filtering. The average mapping percentage was 93.8% (± 2.5%; Data S8).

**FIGURE 3:**
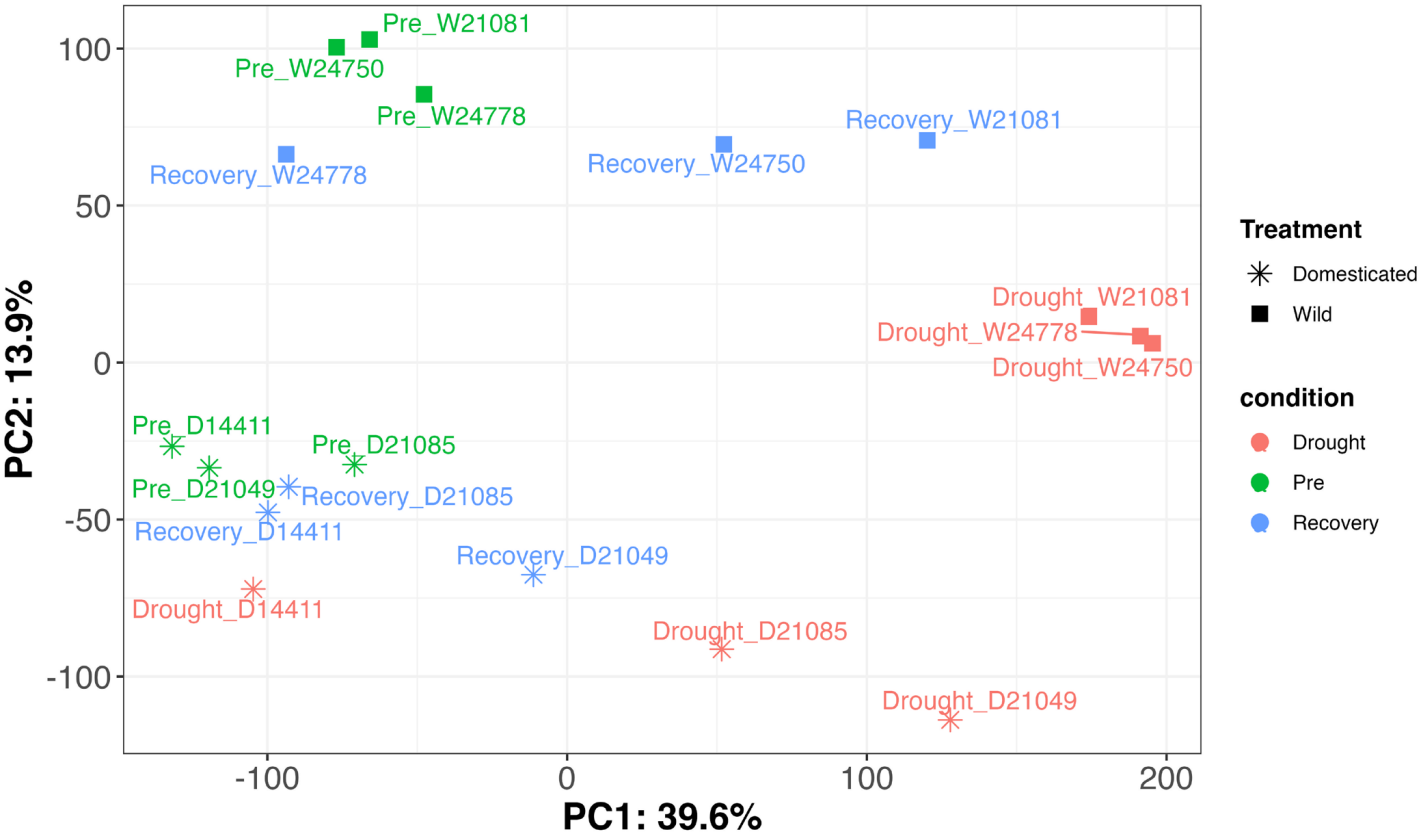
PCA of all RNAseq samples derived from total RNA extracted from the leaves of wild (*) or domesticated (■) accessions of Lablab ( *Lablab purpureus* ) at different stages of drought stress; pre‐drought (green symbols), during drought stress (red symbols) or recovery (approximately 4 weeks after the end of drought stress—blue symbols). Plants were grown in pots for 24 days in a glasshouse before water was withdrawn to initiate drought stress. Water was resupplied after plants showed visible symptoms of drought stress. RNAseq data was generated using paired end sequencing (2 × 150 bp) on an Illumina Novaseq. PCA was generated in R using stats and ggplot libraries.

PCA separated most samples into groups which corresponded to population (wild and domesticated accessions) and timepoints (Figure 3). Two samples do not fully follow this pattern (drought_D14411 and rec_W24778), however they were not removed. PCA2 separated the wild and domesticated accessions, and PCA1 separated the timepoints, with the pre‐drought and recovery samples clustered especially close for the domesticated accessions, suggesting that after the recovery phase, the transcriptome was largely restored to a pre‐drought status.

### 3.3 GO Analysis of Significantly Differentially Expressed Genes

A total of 3915 genes were significantly differentially expressed (padj < 0.05) between pre‐drought and drought timepoints (Table 3). Approximately equal numbers were up‐ and down‐regulated during drought relative to pre‐drought. Among the enriched GO terms were genes associated with terms related to drought and water response such as *response to stimulus*, *cell communication and signaling*, and *stomatal movement* (Data S9a). GO terms involved in reproduction and reproductive processes were also significantly enriched, potentially because accession D21049 flowered during the experiment, which could be evidence of a drought escape strategy.

Within the comparison between pre‐drought and drought timepoints, 3220 genes were significantly differentially expressed between wild and domesticated accessions. Most of these were downregulated in the domesticated compared to the wild accessions and were over‐represented for the GO terms *phosphorus metabolic process*, *cell communication and signaling*, *stomatal movement*, and *response to stimulus* (Data S9b). 457 genes were found to have a significant interaction between timepoint and population (wild vs. domesticated) and were enriched for GO terms *phosphorus metabolic processes*, *cell wall organization*, *response to another organism*, *signaling*, and *auxin transport* (Data S9c; Figure S6).

Only 53 genes were significantly differentially expressed (padj < 0.05) between pre‐drought and recovery timepoints (Table 2). GO analysis revealed that these genes were overrepresented in the following GO terms: *metabolic function*, including *carbohydrate metabolic processes*, as well as *cell communication*, including *regulation of response to stress*, and *response to stimulus*, such as *defense response*, *response to biotic stimulus* (Data S10a).

**TABLE 2:** Statistical analysis of each element based on a 2‐way ANOVA for domestication status, treatment, and interaction. If data were non‐normal and transformed this is indicated in Data S6.

Nutrient	Domestication status (*p*)	Treatment (*p*)	Interaction (*p*)
B (mg/kg)	0.639	0.835	0.199
Ca (g/kg)	0.475	0.894	0.879
Cu (mg/kg)	**0.013**	0.704	0.511
Fe (mg/kg)	**0.002**	0.170	0.231
K (g/kg)	**0.001**	**0.038**	0.684
Mg (g/kg)	0.074	0.073	0.593
Mn (mg/kg)	0.625	0.559	0.294
Mo (mg/kg)	0.077	0.452	0.579
Na (mg/kg)	0.207	0.599	0.442
P (g/kg)	**0.005**	**0.007**	0.282
S (g/kg)	0.760	0.055	**0.001**
Si (mg/kg)	0.288	0.753	0.705
Zn (mg/kg)	**0.000**	0.110	0.257
Nitrogen (%)	0.246	**0.036**	0.835
Carbon (%)	0.438	0.658	0.659
Hydrogen (%)	0.332	0.266	0.237

*Note:* Significant p‐values (*p* < 0.05) for each comparison are highlighted in bold.

**TABLE 3:** The number of significantly differential expressed genes between treatment pairs, between wild and domesticated accessions for each pair of treatments, and their interactions. DE analysis was carried out using DESeq2 v1.37.6 in R.

Data set	Comparison	Total no. of genes in comparison	No. up regulated	% up regulated	No. down regulated	% down regulated	# significant GO terms
a	Pre‐drought vs. drought	21,996	1851	8.42	2064	9.38	588
	Wild vs. domesticated	21,996	890	4.05	2330	10.59	341
	Interaction	21,996	423	1.92	34	0.15	55
b	Pre‐drought vs. recovery	22,056	7	0.03	45	0.20	59
	Wild vs. domesticated	21,880	468	2.14	507	2.32	137
	Interaction	21,880	1	0.00	0	0.00	0
c	Drought vs. recovery	22,056	438	1.99	588	2.67	255
	Wild vs. domesticated	22,038	624	2.83	1555	7.06	248
	Interaction	22,038	76	0.34	5	0.02	32

Within the comparison between pre‐drought and recovery timepoints, 975 genes were significantly differentially expressed between wild and domesticated accessions, with about half upregulated in the wild accessions and the other half in the domesticated. These genes were over‐represented for the GO terms *metabolism*, *immune system processes*, and *responses to stimulus*, including *response to wounding* and *defense response* (Data S10b; Figure S7). Only one gene had a significant interaction.

A total of 1026 genes were significantly differentially expressed (padj < 0.05) between drought and recovery timepoints, with 2.0% of transcripts upregulated and 2.7% downregulated in the recovery timepoint relative to during drought. These genes overrepresented are involved in the following GO terms: *metabolic processes*, *photosynthesis*, *response to stimulus*, *regulation of biological processes* and *oxoacid metabolic process*, which included *vitamin C biosynthetic process* (Data S11a).

Within the comparison between drought and recovery timepoints, 2179 genes were significantly differentially expressed between wild and domesticated accessions. Most of these were upregulated in the domesticated accessions and were over‐represented for the GO terms *anatomical structure development*, *metabolic processes*, *signaling*, *stomatal movement*, and *response to stimulus* (Data S11b). A total of 81 genes were found to have a significant interaction between in the drought and recovery timepoints and the wild versus domesticated analysis and were enriched for GO terms including *carbohydrate metabolism*, *cell wall organization*, *catabolic processes*, *signaling*, and *biogenesis and cell growth* (Data S11c; Figure S8).

### 3.4 Genes Involved in Drought—Known Orthologues

**FIGURE 4:**
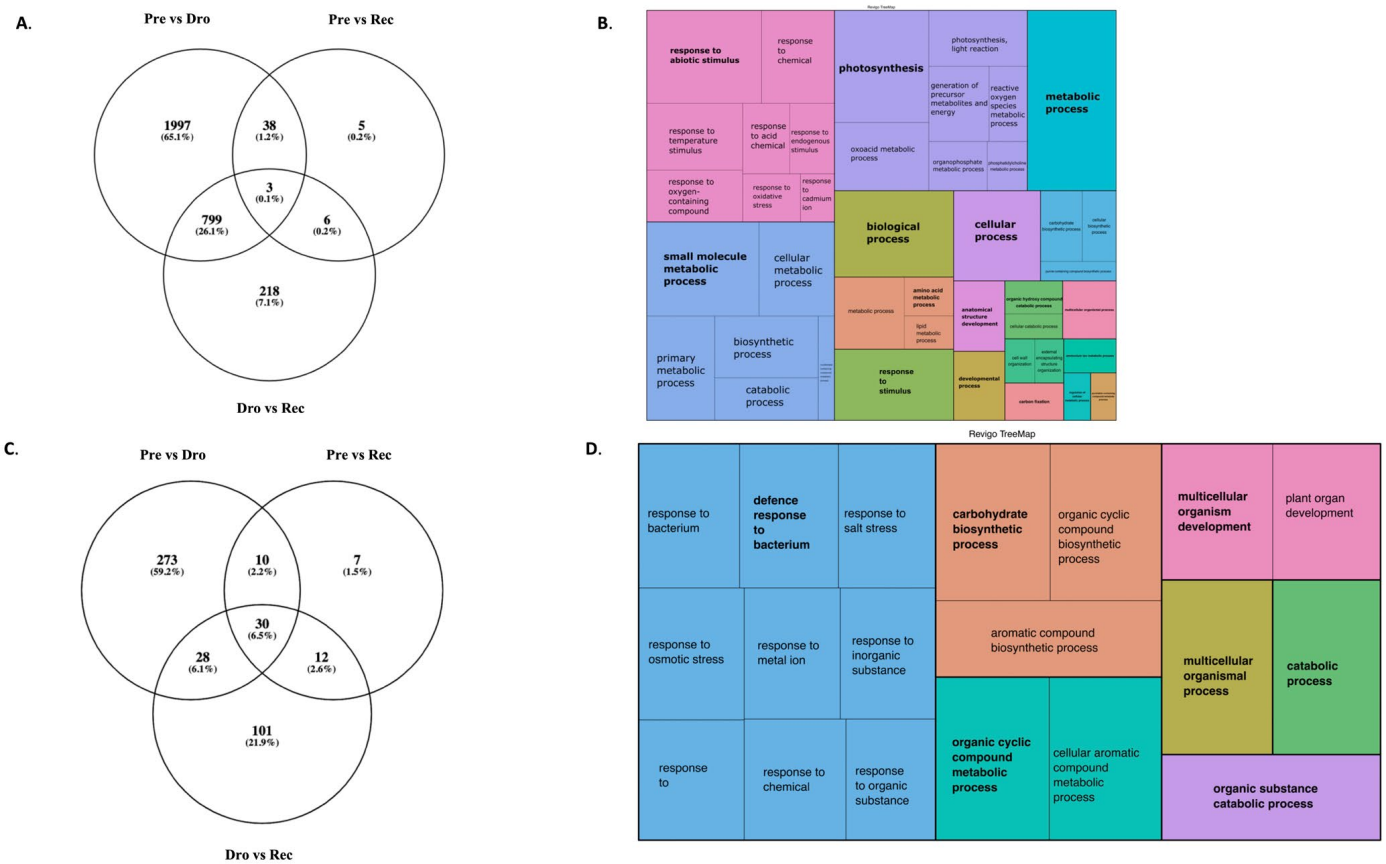
(A) Number of unique and overlapping significantly differentially expressed (DE) genes in the three comparisons (pre‐drought vs. drought (Pre vs. Dro), pre‐drought vs. recovery (Pre vs. Rec) and drought vs. recovery (Dro vs. Rec)); (B) Significantly (adjusted *p* < 0.05) enriched Biological Process GO terms for 799 candidate drought response genes, with the representative terms in bold. The size of box relates to the Log_10_ (P). (C) Number of unique and overlapping GO terms for DE transcripts in the three comparisons (pre‐drought vs. drought, pre‐drought vs. recovery and drought vs. recovery); (D) Significantly (adjusted *p* < 0.05) enriched Biological Process GO terms enriched in 28 GO terms found to be overlapping between the Pre vs. Drought and Drought vs. Recovery datasets, with the representative terms in bold. The size of box relates to the Log_10_ (P).

Drought responsive genes were subsequently defined as those commonly significantly differentially expressed in both pre‐drought versus drought and drought versus recovery comparisons. There was a sizeable overlap (*n* = 799) in the genes which were DE in the pre‐drought versus drought and drought vs. recovery comparisons, with minimal overlap for the other comparisons (Figure 4a). We refer to these 799 as candidate “drought response” genes. Arabidopsis orthologues were identified for these and GO analysis revealed 411 significant (adjusted *p* < 0.05) terms (summarized in Figure 4b). Together, these GO terms come under a small number of groups of terms, for example *response to abiotic stimulus*, *photosynthesis*, and metabolic processes such as *small molecule*, *carbohydrate*, *lipid*, and *ROS metabolism*.

Twenty‐eight GO terms overlapped between the pre‐drought vs. drought and drought versus recovery (Figure 4c). These included *response to stimulus* (including osmotic stress, biotic, chemical stimulus), *plant organ development*, *carbohydrate biosynthetic process*, and *catabolic process* (Figure 4d). These GO terms are similar to the ones found in the overlapping gene dataset.

To understand more about the transcriptional changes under drought stress, we compared the 25 most upregulated and downregulated genes from the overlapping dataset to investigate their functions (Data S12 and S13). The top 25 upregulated genes included a transcription factor that is involved in drought response (AT3G57600) and a protein that integrates ABA. GA, and Glc signaling (AT1G74670/*GASA6*). In addition, there were three genes involved in flowering processes (AT1G54560/*XIE*, AT3G21890/*BBX31*, AT5G62040/*BFT*) and putative disease resistance genes (AT5G17680, AT1G61100).

The top 25 downregulated genes also included ABA‐related genes (AT5G33392/*RD22*, AT5G33386/*NPX1*, AT5G33380/*LTP4*, AT5G33377/*HVA22E*) and three involved in nitrate assimilation (AT5G33382/*NIA1*, AT5G33383/*NIA2*, AT5G33384/*NIR1*).

**FIGURE 5:**
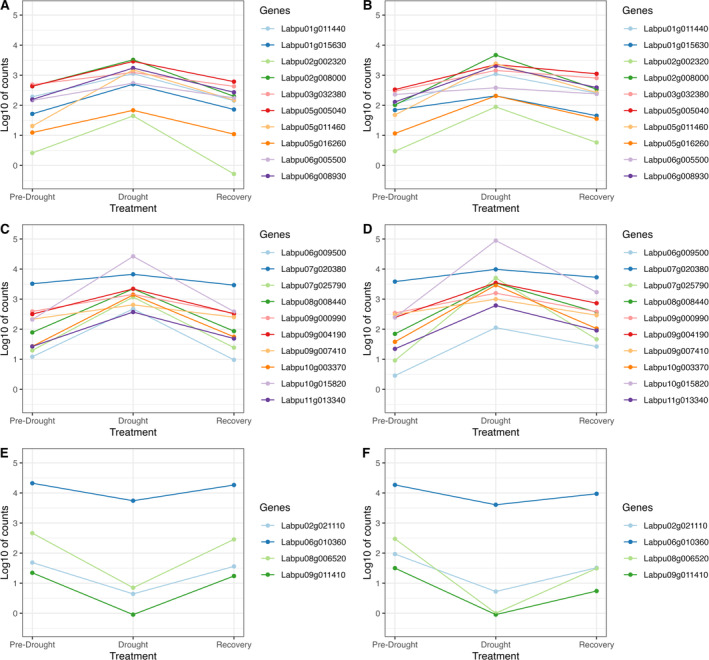
Gene expression dynamics for genes in the *response to osmotic stress* GO category, with (A, C) genes upregulated during drought in domesticated accessions, (B, D) genes upregulated during drought in wild accessions, (E) genes downregulated during drought in domesticated accessions, (F) genes downregulated during drought in wild accessions.

In addition, 24 genes from the GO category *response to osmotic stress* were chosen to investigate the dynamics of gene expression in the experiment. Normalized count data was obtained and averaged separately for the wilds and domesticates (Figure 5; gene names and functions are given in Data S14). For all genes, those upregulated during drought were downregulated in recovery, or vice versa. The same pattern was found in the wild and domesticated groups, however the extent of expression change was not always the same, for example, *Labpu08g006520*, a *HVA22E* homolog, and *Labpu06g009500*, a *HAI1* homolog, are downregulated or upregulated to different extents between the two groups (Figure 5). *HVA22E* is ABA‐ and stress‐ inducible gene upregulated during cold, salt or dehydration stress (Chen et al. 2002). *HAI1* is a negative regulator of osmotic stress and ABA signaling (Zhang et al. 2013).

## 4 Discussion

Our study was designed to investigate how the transcriptome responds to drought in lablab, which is known to be a remarkably drought tolerant legume but remains understudied. In doing so we identify genes and pathways that characterize the drought response, as well as demonstrating how the phenotype (including the leaf element content) is affected by drought and domestication.

### 4.1 Phenotypic and Nutritional Analysis

The number of days taken for the plants to start showing visible signs of drought was not significantly different between the wild and domesticated accessions, with both groups taking around 29 days to become visibly stressed. Leaf water content also did not differ significantly between groups. Leaf biomass, however, was significantly greater (by about 50%–70%) in the domesticated accessions, and was significantly affected by treatment (Figure 1B). Leaf traits are critical for managing water loss and optimizing photosynthesis under drought stress. Drought tolerant plant species, such as cowpea, appear to have a conservative water usage strategy, but are able to maintain higher transpiration rate under drought compared to non‐tolerant soybeans (Fang et al. 2022).

The nutrient analysis demonstrated that the wild and domesticated lablab leaves differ in their nutrient concentration, especially for copper, iron, phosphorus, potassium, and zinc, with the wild accessions having anywhere from 16.90% (iron) to 83.34% (zinc) greater leaf concentrations compared to the domesticated accessions (Figure 2, Figures S1–S4). In comparison with Kabuli chickpea leaves harvested after 33 days, domesticated lablab leaves are on average 22.80% higher in copper, 31.27% higher in phosphorus, but 145.72% lower in potassium, 50.52% lower in iron and 226.97% lower in zinc (Ibrikci, Knewtson, and Grusak 2003). As these ions are important micronutrients for plant and human health, and are commonly consumed by humans in inadequate amounts, this has implications for managing “hidden hunger” (Szabo, Bodolea, and Mocan 2021; Lowe 2021). This may be achieved through either consumption of leaves directly, or through the growth of wild varieties for livestock feed. Lablab leaves increase weight change and average daily gain in lambs, and increased micronutrient intake can support animal productivity in general (Tulu et al. 2024).

Potassium and phosphorus content are affected by both treatment, and domestication status, with droughted wild lablab having the greatest content of these minerals. Most populations in the world consume less potassium than recommended; however global diets are typically sufficient in phosphorus (Farapti et al. 2022; Beal et al. 2017). Phosphorous is important for a variety of bodily functions, including bone health and ATP synthesis, while potassium is necessary for heart and bone health (Serna and Bergwitz 2020; Weaver 2013). The uptake of mineral elements from the soil is contingent on water movement through the soil via mass flow or to facilitate diffusion processes. The greater concentrations of these elements in the leaves of wild lablab accessions may suggest differences in root traits associated with nutrient uptake according to domestication history or due to adaptation to local environment.

Another nutrient affected by treatment was nitrogen content, which was taken as an equivalent to protein content, with drought treated wild accessions having the highest leaf protein concentration, and the wild accessions overall having ca. 1% higher leaf protein concentrations (Figure 2A). Overall, the consumption of wild accessions or breeding domesticated varieties to contain alleles positively affecting these traits could improve people's protein and potassium intake. Understanding these underlying traits will be critical for developing future lablab accessions with high nutritional value under drought stress conditions. Improving protein intake is critical for addressing hidden hunger in a more environmentally friendly way, as it is crucial for development, growth, immune response and more (Safdar et al. 2023). Lablab leaves outperform many commonly consumed leafy vegetables in terms of their nutritional value (Table 4), and therefore are a useful tool for combating malnutrition. While lablab seeds and not leaves are currently used for human consumption, many parts of lablab are edible, and therefore lablab leaves could provide an effective supplement or alternative to leafy greens.

**TABLE 4:** Comparison of non‐droughted lablab to other commonly eaten leafy vegetables, using their raw leaf nutritional values. Lablab data is taken from this study and for the other species nutritional data was obtained from FoodData Central at the USDA (https://fdc.nal.usda.gov/fdc‐app.html#/food‐details/169991/nutrients, accessed December 2023). Data are presented as g/100 g for protein and mg/100 g for micronutrients.

Nutrient	Amaranth	Baby spinach	Kale	Swiss chard	Lablab domesticated	Lablab wild
Protein (g)	2.4	2.85	2.92	1.80	11.88	12.92
Calcium (mg)	215.00	68.00	254.00	51.00	1481.00	1276.00
Iron (mg)	2.32	1.26	1.60	1.80	9.51	12.90
Magnesium (mg)	55.00	92.90	33.00	81.00	583.00	751.00
Phosphorus (mg)	50.00	39.00	55.00	46.00	268.10	324.00
Potassium (mg)	611.00	582.00	348.00	379.00	1697.00	2456.00
Sodium (mg)	20.00	111.00	53.00	213.00	0.87	1.66
Zinc (mg)	0.9	0.45	0.39	0.36	3.82	8.96
Copper (mg)	0.16	0.08	0.05	0.18	1.08	1.32
Manganese (mg)	0.89	0.49	0.92	0.37	10.29	13.31

Overall, while wild lablab leaves seem to have a better nutritional profile compared to domesticated lablabs, the payoff lies in the size of the wild leaves. Wild leaves are smaller than the domesticated leaves, making them an inefficient choice for addressing energy requirements for people or grazing and in our greenhouse experiment, overall biomass was significantly lower per plant than domesticated plants (Figure 1B). However, they may be used as a nutritious supplement alongside other foods that are higher in energy to improve the overall nutrition of a meal. Additionally, wild lablab may prove to be a great reservoir of genetic diversity for the improvement of nutritional qualities in domesticated varieties. However, large‐scale acceptance may be constrained by prevailing consumer attitudes towards lablab, which is often considered to be a “poor man's food” (Hassan and Joshi 2020).

### 4.2 The Overall Genetic Response to Drought in Lablab

According to the GO analysis of drought response genes, lablab drought response involves a complex network of processes, many of which are directly or indirectly related to stress response, including GO terms previously identified in other legumes. The GO annotations uncovered 799 drought response genes included terms involved in general biosynthesis and metabolism, photosynthesis, reactive oxygen species (ROS) metabolism, and response to abiotic stimulus.

GO terms associated with photosynthesis were significantly enriched in the drought response genes, in line with the decreasing photosynthetic efficiency in plants under drought stress. Previous studies from lablab have shown that drought stress is associated with a decrease in chlorophyll fluorescence, while drought tolerance is associated with higher chlorophyll content and stability (Guretzki and Papenbrock 2014). Similar results have been found in other legumes, such as lentils, with drought stress affecting chlorophyll, RuBisCo activity and the photosynthetic rate (Sehgal et al. 2017). An enrichment of GO terms involved in oxidation–reduction processes follows previous findings; Reactive Oxygen Species (ROS) are commonly produced during drought stress, including in legumes (Cruz de Carvalho 2008; Matamoros and Becana 2021).

Hormonal signaling appears to play a key part of the drought response in lablab, as expected from previous studies in legumes (Parwez et al. 2022; Raza et al. 2022). Some of the top up and down‐regulated genes in the drought dataset included ABA‐related genes (Figure 5, Data S11 and S12); ABA is known to participate in the drought response, activating stomatal closure and inducing stress‐related gene expression (Kuromori et al. 2022). An ABA‐responsive gene, HVA22E differed in expression between domesticates and wilds, which may play a role in drought tolerance between domesticated and wild lablab. The pre‐drought vs. drought comparison uncovered GO terms associated with regulation of stomatal movement, with stomatal differences being recorded in previous studies in legumes, including lablab (Guretzki and Papenbrock 2014; Reynolds‐Henne et al. 2010; Sawardekar et al. 2020). Stomatal closure allows the plant to control excessive water loss, and stomatal closure often occurs rapidly during drought (Reynolds‐Henne et al. 2010).

Some of the top down‐regulated genes involved in drought were genes related to nitrogen assimilation. Nitrogen was significantly affected by treatment (*F* = 4.909, *p* = 0.036), increasing in droughted plants, in contrast with previous findings that drought tends to decrease nitrogen assimilation (He and Dijkstra 2014). This appears to be lablab‐specific and has implications in meeting protein requirements for human health. Investigating leaf nitrogen assimilation and symbiotic nitrogen fixation during drought may be done in a future study, as this can enhance production, stress tolerance and lead to the development of effective drought‐tolerant cultivars.

The comparison between pre‐drought and recovery highlighted lablab's exceptional ability to recover quickly from drought, as there were only 52 DEGs in this comparison (compared to > 1000 DEGs in the other comparisons). Some GO terms related to stress and defense response were over‐represented, which is likely due to plants still recovering from drought stress when samples were taken.

Overall, lablab has a similar response to drought to other legumes, suggesting that approaches taken in other plants to boost their drought tolerance may translate to lablab, and vice versa. However, real‐life droughts are likely to be longer than that employed in the experiment and could happen under different conditions, such as higher temperatures, so there is a need for longer‐term field studies to assess drought response and tolerance of wild lablab accessions and those of interest for breeding.

### 4.3 Domestication and Drought Response

Comparisons of gene expression between wild and domesticated accessions during each stage of the drought revealed differences between the two. GO terms related to *cell communication* and *signaling* and *response to stimulus* were significantly enriched in the wild‐domesticated comparisons, which may be connected to metabolic synchronization in the domesticated accessions and local responses in wild plant organs, which can support the plant's ability to respond to environmental changes (Siqueira et al. 2023). In addition, *immune system processes* were a significant GO term in the domesticated and wild accession comparison for the pre‐drought vs. recovery dataset, which could be due to a reduction in plant immunity known to happen with domestication (Singh and van der Knaap 2022).

The interactions comparisons were able to find genes that respond to drought differently in the wild and domesticated populations. GO annotations of the interaction datasets have highlighted a few drought response patterns that differ between wild and domesticated accessions. Both pre‐drought versus drought and drought versus recovery interaction datasets were enriched for GO terms associated with *cell wall organization* and *biogenesis*. This may be due to a reorganization of cell wall structure, which is known to happen during abiotic stress, including drought (Coutinho et al. 2021). This is often because under drought stress, shoot growth needs to be reduced while root growth is maintained (Tenhaken 2014). The way the plants approach this may differ between wild and domesticated accessions. One of the visual differences between the two during recovery was that while domesticated accessions continued growing upwards, the wild accessions often started lateral growth near the base of the stem, while the main shoot and leaves that grew during drought did not develop further.

Drought response of domesticated and wild lablab accessions appears to differ in terms of cellular signaling, as the pre‐drought versus drought dataset is enriched in GO terms to do with cell communication and signaling. GO terms *cell surface receptor signaling pathway* and *transmembrane receptor protein tyrosine kinase signaling pathway* were highly significant, which is likely due to their direct involvement in response to drought, salt and cold stress (Chen et al. 2021). Whether there are parallel transcriptomic responses to other stressors remains to be investigated.

The GO analysis of interaction genes suggests that drought response differs between domesticated and wild accessions in metabolic processes, with *phosphorus metabolic processes* being a significant GO term for the pre‐drought vs. drought interactions dataset. Phosphorus may be able to regulate plant tolerance to abiotic stresses (Khan et al. 2023). Differences in phosphorus metabolic processes can directly affect nutrition, as they will affect how much phosphorus the plants take up. The ability of the accessions to take up phosphorus may be affected by their root traits. Going forward, assessing root traits and transcriptomes could be explored to further elucidate drought response. Therefore, wild and domesticated lablabs show a difference in stress response, but the precise molecular differences between their strategies remain to be elucidated.

## 5 Conclusion

With extreme weather conditions increasing globally, there is a pressing need to help our food systems adapt to new pressures and challenges. Rediscovering and improving underutilized crops may be part of the solution. This study explored the response to and recovery from drought stress of domesticated and wild *Lablab purpureus* accessions. Wild and domesticated lablab respond similarly to drought and as other legumes. Our study is the first that investigates how lablab nutrient content is affected by drought, showing that wild lablab contains a greater nutrient concentration and that some key nutrients are affected by drought, offering breeding targets for the future.

Our data supports further development of lablab as a key crop in improving food security in a changing climate. Exploring seed nutrients and anti‐nutrients after exposure to drought are a major target for future research, as lablab seeds are the most commonly consumed part of the plant, and the root traits that support both nutrient acquisition and tolerance to drought. In addition, further exploration of the existing lablab diversity in drought response may further assist breeding and commercialization. Additionally, consumers may be hesitant to include underutilized crops in their diet, as these have culturally been seen as “poor man's food” and so cultural awareness needs to be addressed.

## Conflicts of Interest

The authors declare no conflicts of interest.

## Supporting information


Supplementary Figures S1‐S8.

Supplementary Datasets S1‐S14

## Data Availability

All phenotypic data relevant to the study are available in the Supporting Information. All relevant sequencing data has been deposited to the SRA under BioProject: PRJNA1153959.
