# Supplementary material for: Drought Response in the Transcriptome and Ionome of Wild and Domesticated Lablab purpureus L. Sweet, an Underutilized Legume
Source: Plant Environ Interact. 2025 Jan 19;6(1):e70027. doi: 10.1002/pei3.70027 (PMC11742185; doi:10.1002/pei3.70027)
Supplement: Supplementary file 1 — Supplementary Figures S1‐S8. [file PEI3-6-e70027-s002.docx]

## Plant and Environment Interactions Supporting Information

Article title: Drought response in the transcriptome and ionome of wild and domesticated *Lablab purpureus*, an underutilised legume.

Authors: Anastasia Kolesnikova, John Hammond, Mark A. Chapman

The following Supporting Information is available for this article:

**Fig. S1.** Lablab plants undergoing drought stress. A. Yellowing of leaves in a domesticated lablab accession. B. Drooping of leaves in a domesticated lablab accession. C. Yellowing of leaves in a wild lablab accession. D. Drooping of leaves in a wild lablab accession.

**Fig. S2.** Nutritional content in wild and domesticated lablab leaves under different conditions. Error bars indicate standard error. A. Mean leaf carbon; B. Mean leaf hydrogen.

**Fig. S3.** Nutritional content in wild and domesticated lablab leaves under different conditions. Error bars indicate standard error. A. Mean leaf boron content; B. Mean leaf calcium content; C. Mean leaf copper content; D. Mean leaf magnesium content.

**Fig. S4.** Nutritional content in wild and domesticated lablab leaves under different conditions. Error bars indicate standard error. A. Mean leaf manganese content; B. Mean leaf molybdenum content; C. Mean leaf sodium content; D. Mean leaf sulphur content. Significant comparisons found by a TukeyHSD test and their p-values are represented by a bracket.

**Fig. S5.** Nutritional content in wild and domesticated lablab leaves under different conditions. Error bars indicate standard error. A. Mean leaf silicon content; B. Mean leaf iron content. Significant comparisons found by a TukeyHSD test and their p-values are represented by a bracket.

**Fig. S6.** Revigo tree map summarizing key GO terms in Biological Process for interactions dataset in the pre-drought vs drought comparison.

**Fig. S7.** Revigo tree map summarizing key GO terms in Biological Process for the wild vs domesticate comparison in the pre-drought and recovery dataset.

**Fig. S8.** Revigo tree map summarizing key GO terms in Biological Process for the interactions dataset in the drought and recovery comparison.

A. B.


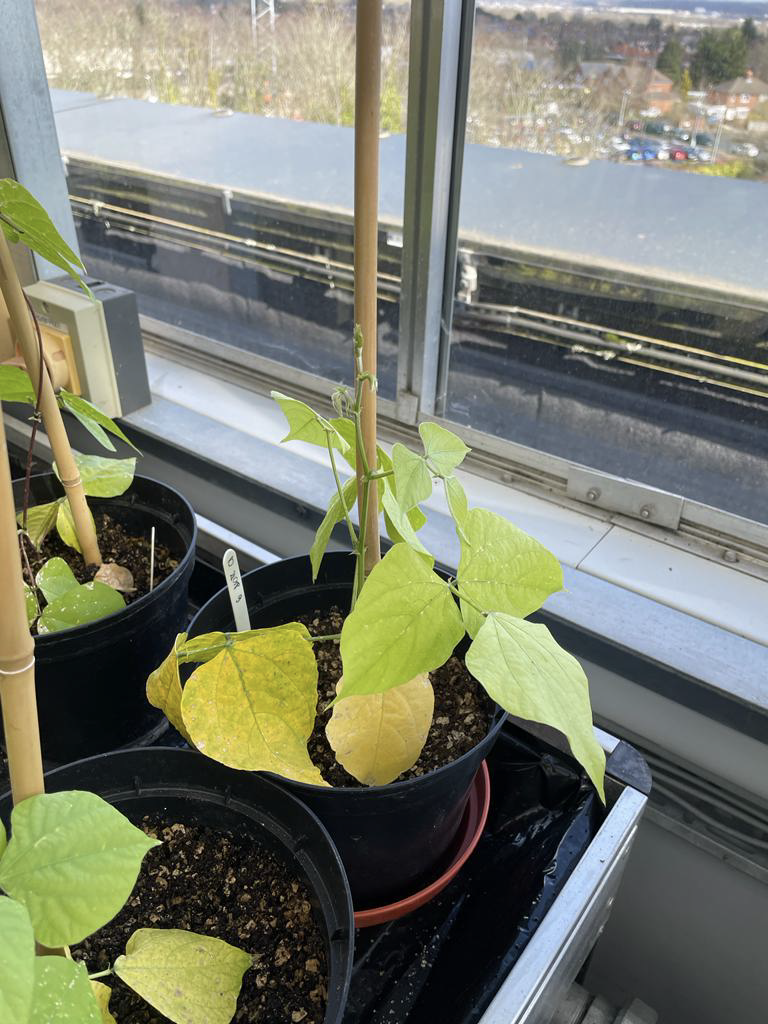

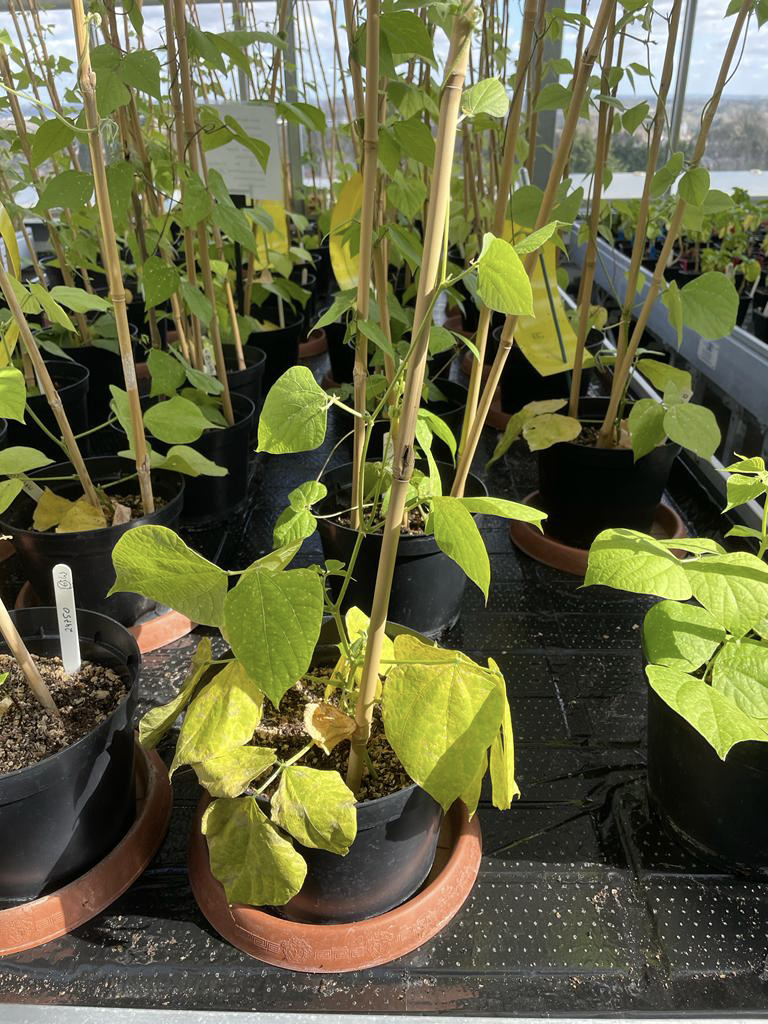


C. D.


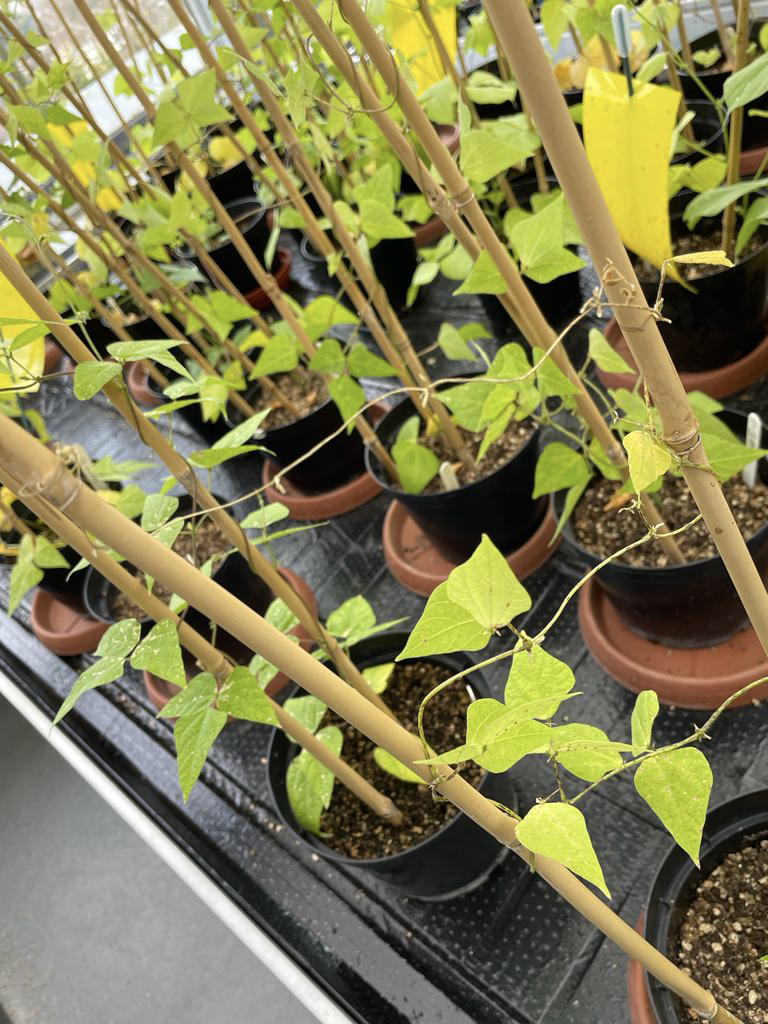

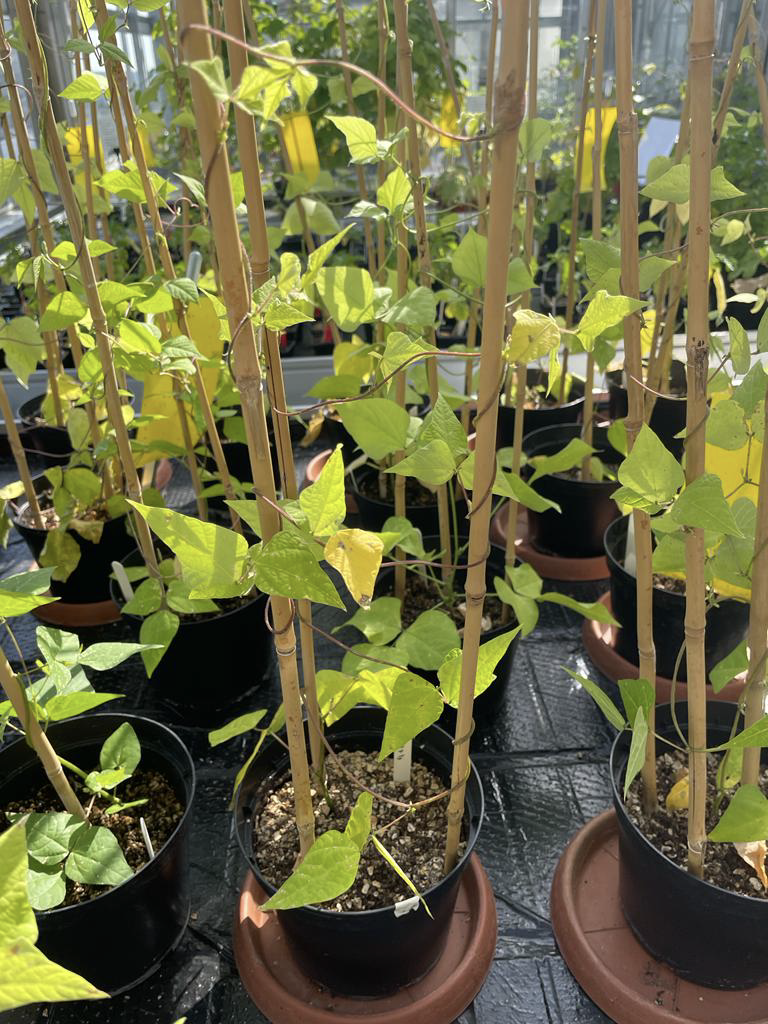


**Fig. S1.** Lablab plants undergoing drought stress. A. Yellowing of leaves in a domesticated lablab accession. B. Drooping of leaves in a domesticated lablab accession. C. Yellowing of leaves in a wild lablab accession. D. Drooping of leaves in a wild lablab accession.


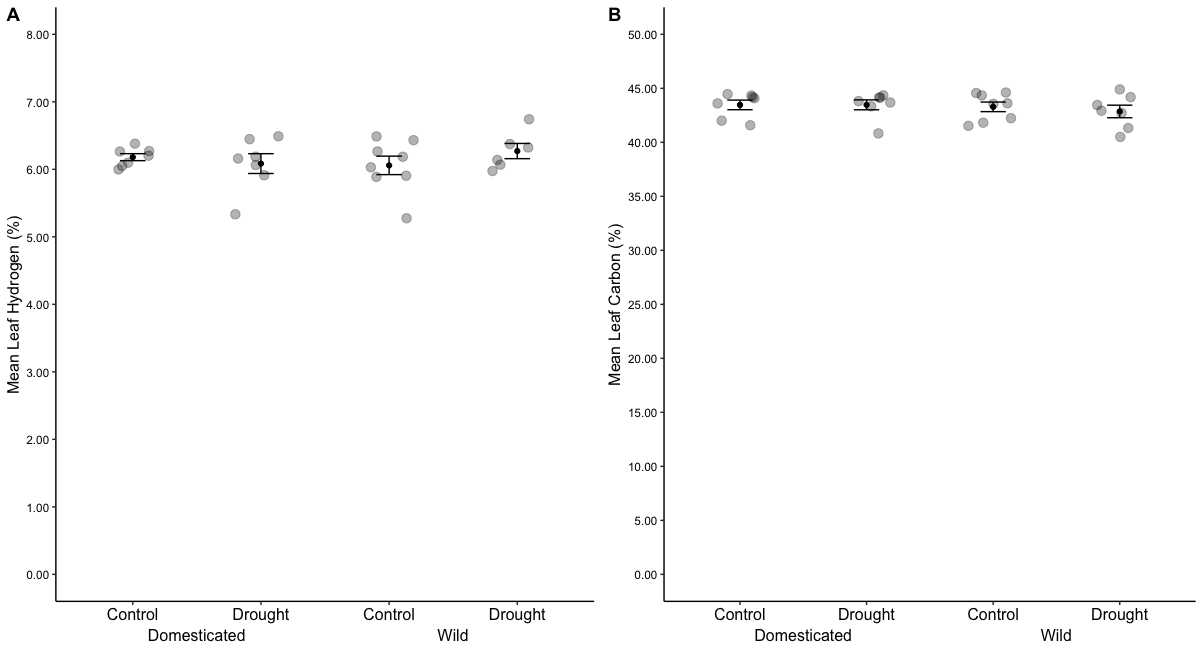


**Fig. S2.** Nutritional content in wild and domesticated lablab leaves under different conditions Error bars indicate standard error. A. Mean leaf carbon; B. Mean leaf hydrogen.


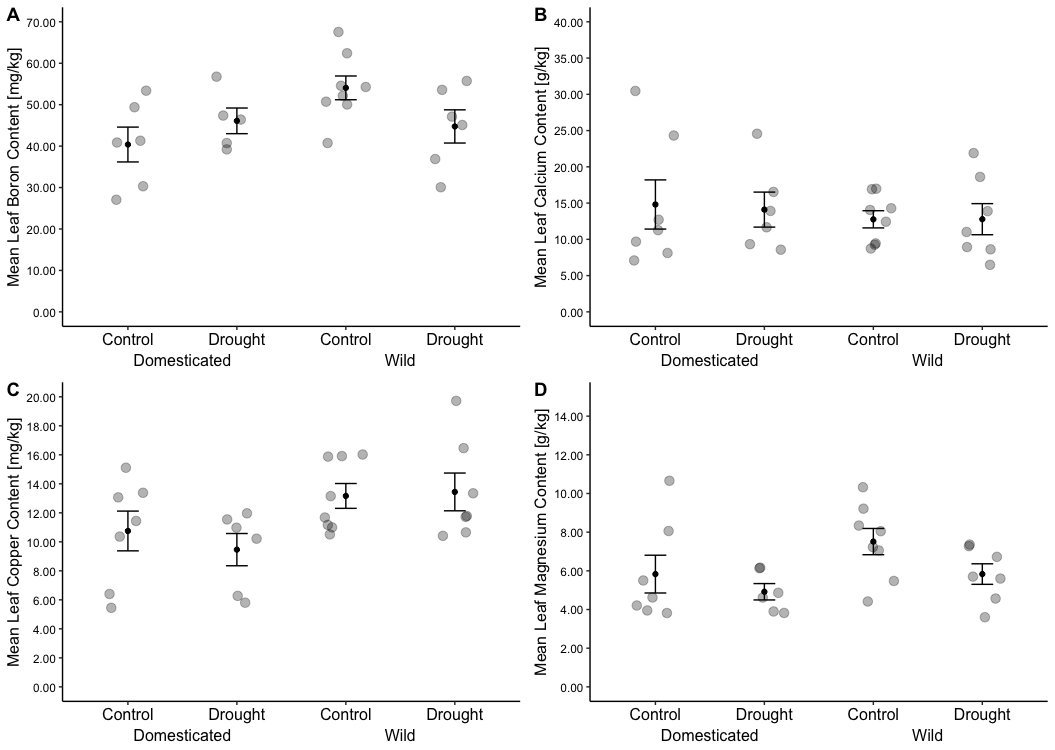


**Fig. S3.** Nutritional content in wild and domesticated lablab leaves under different conditions. Error bars indicate standard error. A. Mean leaf boron content; B. Mean leaf calcium content; C. Mean leaf copper content; D. Mean leaf magnesium content.

**
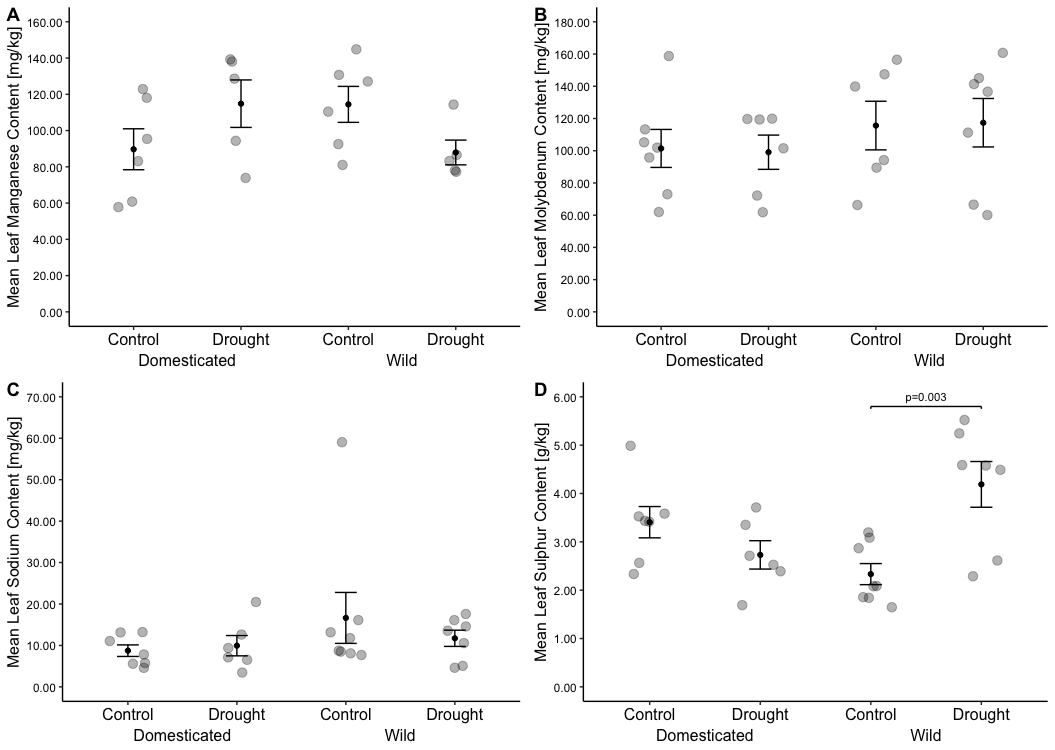
**

**Fig. S4.** Nutritional content in wild and domesticated lablab leaves under different conditions. Error bars indicate standard error. A. Mean leaf manganese content; B. Mean leaf molybdenum content; C. Mean leaf sodium content; D. Mean leaf sulphur content. Significant comparisons found by a TukeyHSD test and their p-values are represented by a bracket.


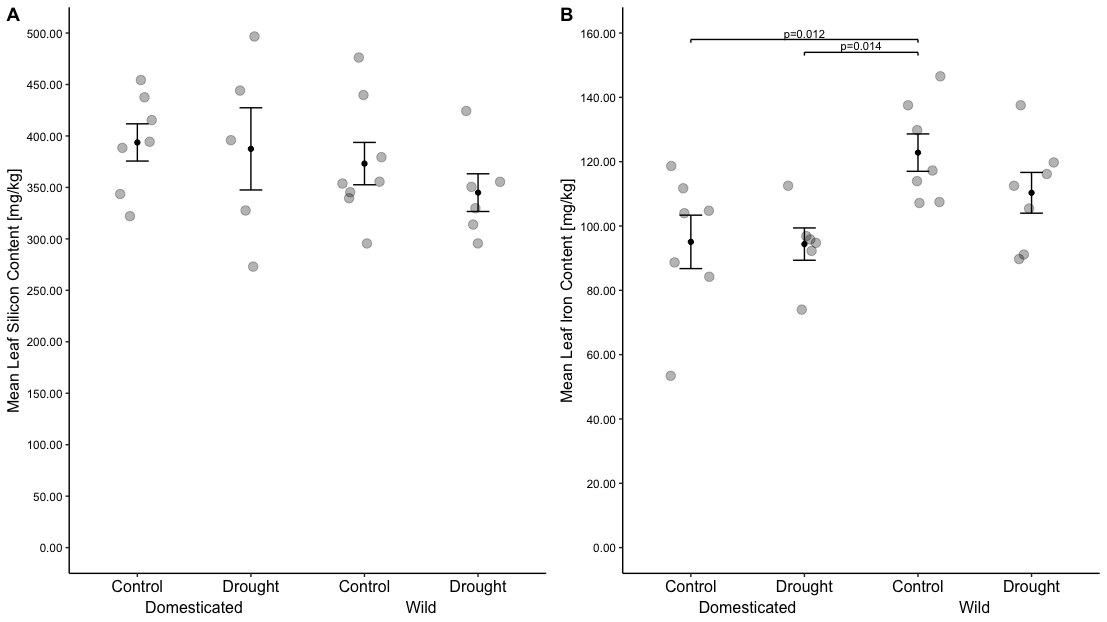


**Fig. S5.** Nutritional content in wild and domesticated lablab leaves under different conditions. Error bars indicate standard error. A. Mean leaf silicon content; B. Mean leaf iron content. Significant comparisons found by a TukeyHSD test and their p-values are represented by a bracket.


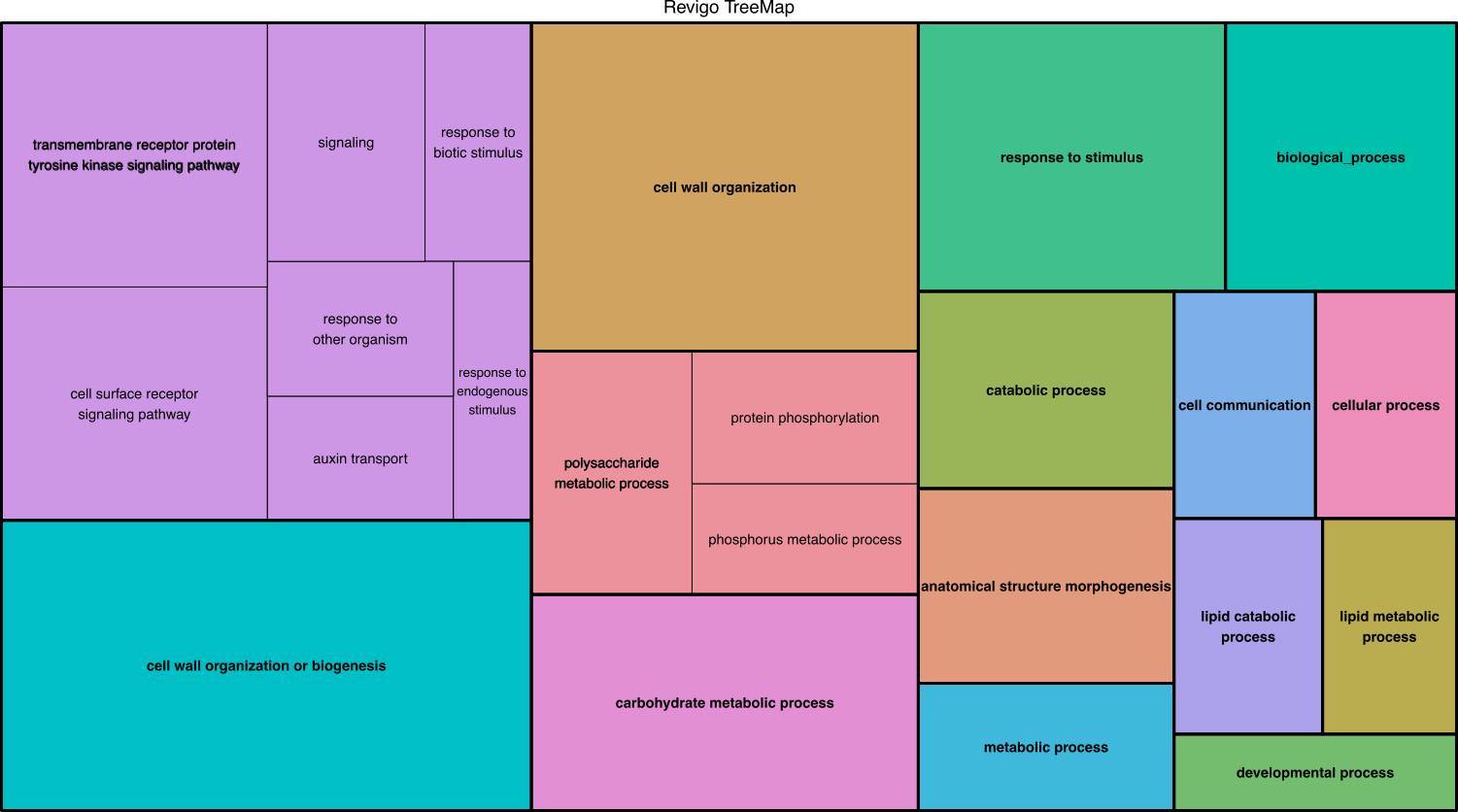


**Fig. S6.** Revigo tree map summarizing key GO terms in Biological Process for interactions dataset in the pre-drought vs drought comparison.


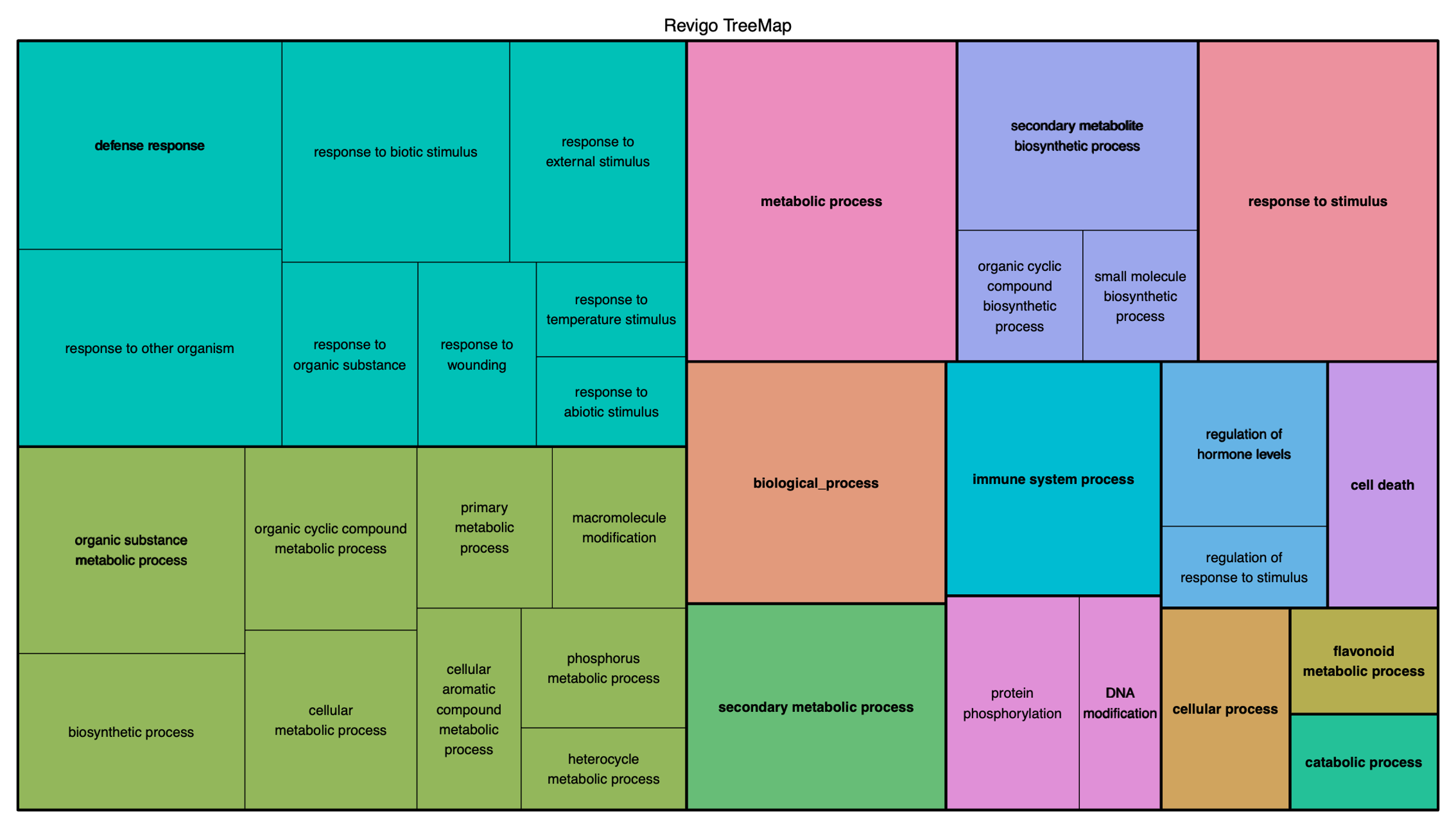


**Fig. S7.** Revigo tree map summarizing key GO terms in Biological Process for the wild vs domesticate comparison in the pre-drought and recovery dataset.

**Fig. S8.** Revigo tree map summarizing key GO terms in Biological Process for the interactions dataset in the drought and recovery comparison.
